# Intra-community infanticide in wild, eastern chimpanzees: a 24-year review

**DOI:** 10.1007/s10329-019-00730-3

**Published:** 2019-05-27

**Authors:** Adriana E. Lowe, Catherine Hobaiter, Caroline Asiimwe, Klaus Zuberbühler, Nicholas E. Newton-Fisher

**Affiliations:** 1 Living Primates Research Group, School of Anthropology and Conservation, University of Kent, Canterbury, UK; 2 Centre for Social Learning and Cognitive Evolution, School of Psychology and Neuroscience, University of St Andrews, St Andrews, UK; 3 Budongo Conservation Field Station, Masindi, Uganda; 4 Institute of Biology, University of Neuchatel, Neuchâtel, Switzerland

**Keywords:** Infanticide, Aggression, Sexual selection, *Pan troglodytes*, Budongo

## Abstract

Infanticide is well documented in chimpanzees and various hypotheses have been proposed to explain this behavior. However, since infanticide by chimpanzees is relatively rare, it has thus far not been possible to thoroughly test these hypotheses. Here we present an analysis of the largest dataset of infanticides from a single community of chimpanzees, a full record of all intra-community infanticides and failed attempts at infanticide over a 24-year period for the Sonso community of chimpanzees in the Budongo Forest, Uganda. We use these data to test four hypotheses for this behavior: the sexual selection hypothesis, male mating competition, resource competition, and meat acquisition. Our dataset consisted of 33 attacks on 30 victims, 11 of which were ‘definite’ infanticides, four of which ‘almost certain’, and nine were ‘suspected’, while nine were ‘attempted’ infanticides. The majority of attacks where the perpetrators were known (23) had only male attackers and victims were disproportionately young (two-thirds of victims with known ages were under 1 week old). Our data best support the sexual selection hypothesis for infanticide. Cannibalism was infrequent and partial, suggesting meat acquisition was a by-product of infanticide, and there was no evidence to suggest that infanticide was part of a male strategy to eliminate future competitors. Infanticide by females was rare, but we suggest sexual selection, operating through intra-sexual competition, may also be responsible for infanticide by females.

## Introduction

Intra-community infanticide is well documented in East African chimpanzees (*Pan troglodytes schweinfurthii*). In a 2014 review of 12 eastern (*P. t. schweinfurthii*) and six western (*P. t verus*) populations of chimpanzees, as well as four bonobo (*Pan paniscus*) populations, 45 observed, inferred, or suspected intra-community infanticides by chimpanzees were reported (Wilson et al. 2014). Of these, only one was from a West African population. No cases were reported for the bonobos. Since then, one further East African case has been reported in the literature (Nishie and Nakamura 2018), although it is likely that there have been other unreported incidences in the intervening years: 17 of the 45 cases complied by Wilson et al. (2014) were drawn from previously unpublished data. Given chimpanzees’ high fission–fusion social dynamics, the lower gregariousness of females compared to males (Wrangham 2000) and the spatial dispersion of females’ core areas, researchers almost certainly do not see all cases, particularly if they occur soon after birth. Infanticide is likely to be an important selective pressure, given the slow reproductive rate of chimpanzees and the substantial energetic costs for mothers of pregnancy and lactation (Murray et al. 2009; Emery Thompson 2013); thus, detailed information on the nature of infanticidal attacks is particularly valuable.

The data on intra-community infanticide in chimpanzees does not currently support a single, adaptive explanation for this behavior, potentially because of the lack of detailed reports. Here we discuss four distinct, previously proposed hypotheses for infanticide in chimpanzees: the sexual selection hypothesis (Hrdy 1979; Sommer 1987; van Schaik 2000), male mate competition (Takahata 1985), resource competition (Hrdy 1979), and meat acquisition (Hrdy 1979) in the light of existing evidence (compiled by Wilson et al. 2014). The first and second of these propose explanations drawn from sexual selection; the other two propose natural selection explanations. For full details of the predictions of each hypothesis, see Table 1. We then use 24 years of data from a single, highly infanticidal population of chimpanzees from the Budongo Forest, Uganda, to test these hypotheses. While we evaluate each hypothesis independently, we recognized that they are not necessarily mutually exclusive: multiple adaptive benefits may have selected for infanticidal behavior, whether simultaneously or sequentially, and some benefits may be incidental by-products.

**Table 1:** Summary of the predictions of the four hypotheses for infanticide

	Sexual selection	Resource competition	Meat acquisition	Mate competition
Age bias in victims	Yes, younger	–	No	–
Killers should not target their own offspring	Yes	–	–	–
Killers should sire replacement offspring	Yes	–	–	–
Sex bias in attackers	Yes, male	Yes, female	Yes, female	Yes, male
Sex bias in victims	No	No	No	Yes, male
Mothers should be uninjured	Yes	–	–	–
Victims will be cannibalized in full	–	–	Yes	–
Killers should eat the carcass	–	–	Yes	–

*Dashes* indicate where the hypothesis does not yield a specific prediction

The sexual selection hypothesis (Hrdy 1979; Sommer 1987; van Schaik 2000) makes several predictions. As lactation prevents females from ovulating (McNeilly et al. 1994), dependent infants are an obstacle for males seeking reproductive opportunities. A younger infant is a larger obstacle, as it represents a longer period until the mother will be sexually receptive again. This cost is quantified in van Schaik (2000)’s model for sexually selected infanticide, in which *B* (benefit for the perpetrator of committing infanticide) is the reduction in time until the mother is free to mate again that a male could bring about by killing her present infant. *B* will be largest when infants are youngest, and therefore this hypothesis predicts that the majority of infants killed by infanticide should be very young (infants < 1 year). In addition to infants being very young [sexual selection hypothesis prediction one (S1)], killers should not kill their own infants (prediction S2), and should often father replacement infants (prediction S3). Attackers should be males (prediction S4), and victims should be neither predominantly male nor female since both represent an equal obstacle to mating with the mother (prediction S5). Mothers should not usually sustain severe injuries (prediction S6): they are not the primary targets, and severely injuring a female may compromise future mating opportunities. Existing data provide mixed support for sexually selected infanticide. Evidence supporting this hypothesis has been reported for a variety of primate species (Daly and Wilson 1984; Watts 1989a; Borries et al. 1999; Palombit et al. 2000), but while some have argued that the sexual selection hypothesis explains within-community infanticide in chimpanzees (for example: Otali and Gilchrist 2006; Murray et al. 2014, 2016), any adaptive benefits should be reduced in species with multi-male groups, such as chimpanzees, due to both higher paternity uncertainty making it more likely that a male will kill his own infant, and competition for any mating opportunity created. The sexual selection hypothesis has not yet been tested explicitly for chimpanzees, although male infanticide has been documented in several primate species which live in multi-male groups (for example: red colobus (*Piliocolobus badius*): Struhsaker and Leland 1985; Hanuman langurs (*Semnopithecus entellus*): Borries 1997; chacma baboons (*Papio ursinus*): Palombit et al. 2000).

Of the 35 intra-community infanticides reported by Wilson et al. (2014), excluding those from our study site (the Budongo Forest Reserve, Uganda), in eight instances the attackers were male, in six instances they were female, while both sexes were attackers in two instances; the sex of the attackers was unknown in the remaining 19 cases. The mean age of victims where age was known (31 cases) was 0.46 years (SD 0.79), so infanticide would have shortened the mother’s period of lactational amenorrhoea by several years (average weaning age 4.5–5.5 years: Pusey 1983; Bădescu et al. 2017). Male infants were killed more frequently than female infants (19 versus seven incidences, respectively), although these data were not adjusted for potential short-term variation in the birth sex ratio. Mothers were often unharmed, even when the infants were taken in circumstances where the attackers seemed capable of killing them as well (Wilson et al. 2014). Based on these existing data, predictions S1 and S6 (infants are young and mothers are often left unharmed) are supported, but not predictions S4 and S5 (no evidence of sex bias in attackers, and a bias towards male victims, although this may not be statistically significant and in many cases data on attacker or victim sex are not available). Data on paternity are often lacking in cases of infanticide, partly due to the difficulty of retrieving corpses for analysis. Infants were deemed unlikely to be, or definitely not, sired by attackers in some cases (Murray et al. 2007; Nishie and Nakamura 2018) but attackers were considered possible fathers in others (Nishida and Kawanaka 1985; Wilson et al. 2004). Data on the proportion of cases where killers sire subsequent offspring with the mothers of their victims are also lacking, so there is not enough evidence to evaluate predictions S2 and S3.

The second sexual-selection-based hypothesis proposed to account for infanticide in chimpanzees is male mating competition (Takahata 1985). While females should compete over food, male fitness is primarily constrained by access to females (Bateman 1948), making fertile females their most valuable resource. Males could potentially target male infants who will one day be competitors for this resource, and this has been proposed as an explanation for infanticide in both chimpanzees (Takahata 1985) and langurs (Agoramoorthy and Mohnot 1988). This hypothesis generates the predictions that infanticides should be committed by males (male mating competition hypothesis prediction one (MC1), and that they should target male infants (prediction MC2). Of the eight male-committed intra-community infanticides reported for chimpanzees at sites other than Budongo, five involved male victims and one had a female victim, while the sex of two victims was unknown (Wilson et al. 2014). While these data are not inconsistent with the hypothesis, the attacking of the female infant by adult males, and infanticidal attacks by females, require alternative explanations. We leave the issue of whether the strategic cost of reducing the total number of males within a community makes sense, given territorial pressure from neighboring communities, for the discussion.

The third hypothesis that we consider here is the resource-competition hypothesis, which proposes that infanticidal individuals target infants who are competitors for resources (Hrdy 1979). Infanticide is therefore the consequence of natural selection. In order for such killing to be adaptive, it must result in greater access to resources, such as food, for the killer and/or their kin (Hrdy 1979). Resource-competition motivated infanticide is often described between females (e.g., in Belding’s ground squirrels (*Urocitellus beldingi*), infanticidal females can gain access to better nest sites by killing the young of other females who then abandon their ‘unsafe’ burrows: Sherman 1976). As female fitness is linked to resource quality in at least one community of chimpanzees (Emery Thompson et al. 2007c), and the potential for female competition is high (e.g., at Gombe: Pusey and Schroepfer-Walker 2013), females may benefit by removing competitors depending on ecological circumstances. Female-committed, resource-competition-motivated infanticide in chimpanzees would be typified by a pattern of female attackers [resource competition hypothesis prediction one (R1)] and no bias in the sex of victims (prediction R2) since both are equal competitors for food. Of the reported cases of infanticide at all sites excluding our study community, there was no sex bias in attackers: six attacks were by females only, eight by males only and two had attackers of both sexes (Wilson et al. 2014). While twice as many attacks had male victims (eight vs. four: Wilson et al. 2014), females were no more likely to attack male infants than female infants (female only attackers killed two male and two female infants, as well as two of unknown sex: Wilson et al. 2014). Thus, resource competition is unlikely to account for the infanticides committed by male chimpanzees, but may be a factor in female-committed infanticides.

As well as being a potential competitor for resources, infants could be the resource (Hrdy 1979). Meat is an important source of protein for chimpanzees and may provide necessary micronutrients (Tennie et al. 2009; Fahy et al. 2014). Hunting is not always successful and success rates vary between communities, from 42% in the Sonso community (Hobaiter et al. 2017), 52% at Gombe (Stanford et al. 1994), 60% at Mahale and Taï (Boesch and Boesch 1989; Hosaka et al. 2001) and 72% at Ngogo (Watts and Mitani 2002). Variation in hunting frequency within communities is correlated with fruit abundance (Mitani and Watts 2001; Gilby and Wrangham 2007), indicating that it involves an energetic cost, and since unsuccessful hunts yield no compensatory benefits, opportunistically snatching conspecific infants may be a comparatively low-cost way of obtaining meat. The meat acquisition hypothesis predicts that the carcasses of victims will always be eaten and eaten in their entirety, as the primary motivation will be to acquire meat [meat acquisition hypothesis prediction one (M1)]. In addition, the killer should always be the one to eat (or at least among those who eat) the carcass, if cannibalism is the underlying cause (prediction M2). The meat acquisition hypothesis does not predict a bias in sex or age of the victims, unlike in hypotheses such as sexual selection where infant age is a key factor. Younger infants may be easier to snatch as they are less able to defend themselves, but chimpanzees regularly hunt *Pilocolobus tephreosceles* and *Colobus guereza* which reach up to 13 and 23 kg, respectively (Stanford et al. 1994; Kingdon 1997; Newton-Fisher et al. 2002), and while infant and juvenile monkeys are preferred, chimpanzees are capable of catching and killing adult monkeys of these species and adults make up 25% of captures at Gombe (Stanford et al. 1994). As a 5-year-old chimpanzee weighs an average of ~ 20 kg (Grether and Yerkes 1940), adults should be able to prey on them throughout the infancy period. In addition, larger infants may be preferred as they are less likely to be in direct contact with the mother and would make a more substantial meal. The main factor in selecting a victim would most likely be the opportunity to snatch it from the mother, meaning that victims should be a mix of different ages.

In most communities, female chimpanzees are less likely to be actively involved in hunting and consequently eat less meat (Mitani and Watts 2001; Watts and Mitani 2002; Reynolds 2005; Gilby et al. 2017; but see also: Pruetz and Bertolani 2007; Newton-Fisher 2015). Watts and Mitani (2002) reported only five kills by females of prey animals (red and blue duiker, red colobus) during a 32-month observation period in which 131 predation episodes were witnessed, indicating that females could particularly benefit by targeting young conspecifics as an alternative source of meat. If meat acquisition is the primary motivation for infanticide in chimpanzees, attackers should be predominantly females (prediction M3) as they have reduced access to other meat sources, and, unlike males who do not know which infants they have fathered, will not risk accidentally killing their own offspring when targeting infants as a food resource. This hypothesis does not preclude the possibility of attacks by males, but attacks should be primarily perpetrated by those individuals who have fewer alternative sources of meat. Cannibalism is well documented in infanticide cases (Goodall 1977; Takahata 1985; Newton-Fisher 1999; Watts and Mitani 2000; Walker et al. 2018a; Kirchhoff et al. 2018; Nishie and Nakamura 2018), and is the norm in at least two populations (12 of 14 within-group incidences in the Kanyawara community, Kibale, Uganda: Arcadi and Wrangham 1999, and all cases in which the infanticide was successful at Gombe, Tanzania: Murray et al. 2007). However, infanticide victims at Gombe (the only site for which published data exist) tend to be exploited less fully than prey animals, with less nutritionally rich areas (such as hands and feet, rather than organs or large muscle groups) more likely to be eaten (Kirchhoff et al. 2018). Infanticide without cannibalism has also been reported, including at Budongo (Arcadi and Wrangham 1999; Townsend et al. 2007). While a lack of a sex bias in victims supports this hypothesis, the bias towards younger victims and the frequency of male attacks (Wilson et al. 2014), as well as the pattern of consumption, suggests that meat acquisition is unlikely to have been the primary selective factor shaping infanticidal behavior in chimpanzees.

In this study, we use new data on intra-community infanticides in a population of wild East African chimpanzees to further test these hypotheses. The combined evidence from other research sites does not currently offer strong support for any of the four proposed adaptive explanations for infanticide in chimpanzees, and we use the largest dataset on infanticide from any single community to date to address this gap in our understanding and to look more closely at the function of this adaptively significant behavior.

## Materials and methods

### Data collection

The Budongo Conservation Field Station (BCFS, previously the Budongo Forest Project) has collected behavioral data on the Sonso community of chimpanzees since 1993 (Plumptre 1996; Newton-Fisher 1997; Reynolds 2005), alongside demographic information tracking births, deaths, immigrations, and emigrations. The Sonso community’s home range comprises ~ 7 km^2^ of moist, medium altitude, semi-deciduous rainforest within the 428 km^2^ Budongo Forest Reserve (Eggeling 1947; Newton-Fisher 1997, 2003). During the study period (1993–2017), the Sonso community consisted of 34–71 individuals (median: 60). The number of adult males (≥ 16 years) ranged from 5 to 15 (median 10) and the number of adult females (≥ 14 years) ranged from 16 to 28 (median 23). Ages of individuals who were adults when data collection began were estimated by comparison to known-age individuals in other habituated communities, particularly the Kasakela community, Gombe National Park, Tanzania (Newton-Fisher 1997; Reynolds 2005), as were those of females who immigrated into the community. Infant ages were calculated from the birth date when known, or the midpoint of a date range when the exact date was unknown.

Data analyzed here were collected by field assistants and researchers studying this community between 1993 and 2017. Field assistants, the veterinary team, and researchers recorded unusual or rare events in a series of log books maintained by the field station. While such qualitative reports form an imperfect record, infanticides are an event of sufficient rarity and interest that they are typically recorded in considerable detail. We used these records to compile as comprehensive a history as possible of intra-community infanticides among the Sonso chimpanzees. We collated all accounts of infanticide and extracted, for each case: date, identity, age and parity of the mother, age and sex of the infant at the time of the attack, injuries sustained, whether the attack was successful or not (i.e., did the infant survive), whether the infant was cannibalized, age and sex of the attacker(s), injuries to the mother, and whether or not other community members defended the victim.

Long-term demographic records from BCFS show 103 infant births, and 50 deaths or disappearances of individuals aged under 5 years, since data collection began (1993–2017). Of these 50 cases, we excluded four where uncertainty around birth and/or disappearance dates made it possible that they were no longer infants at the time of disappearance, leaving a dataset for analysis of 46 cases. Given the fission–fusion dynamics of chimpanzee social organization, peripheral females may be seen only rarely, and we recognize that some births, and possibly infanticides, may be missing from our dataset.

### Data analysis

#### Calculating frequency of intra-community infanticide

We categorized each death/disappearance according to the likelihood that it was due to an infanticidal attack. Only those incidences when the moment of death, fatal wounding, or permanent separation from the mother was observed, were listed as definite infanticides. When attacks were observed and infants disappeared or died days later, these were listed as ‘almost certain’. We considered incidences in which infants had severe injuries to be indicative of infanticide, particularly when those injuries were unlikely to be sustained by a fall and were similar to those sustained in observed attacks (e.g., bite marks, disembowelling). These were listed as ‘almost certain’ or ‘suspected’ depending on the injuries sustained and behavior of other individuals in the vicinity. Leopards (*Panthera pardus*) are absent from the study area (Schel and Zuberbühler 2009) and crowned-hawk eagles (*Stephanoaetus coronatus*), despite killing monkey prey of up to ~ 11 kg, appear not to prey on chimpanzee infants (Sanders et al. 2003). Predation pressure is therefore low in the Sonso community and injuries that are not consistent with a fall (e.g., limbs or scalp torn off) are likely to be inflicted by conspecifics. Where mothers were seen carrying dead infants but no attack was observed, this was categorized as ‘suspected’ if an infanticidal attempt had been observed in the previous days. We consider the majority of ‘unknown’ deaths to be relatively unlikely to be infanticides: Sonso community chimpanzees are observed by researchers and field assistants almost every day, their home range is relatively small, and most observed infanticide events have included multiple, loud vocalizations and substantial commotion. Field assistants and researchers typically abandon regular data collection in order to investigate when the chimpanzees’ vocalizations indicate an unusual event and are likely (although not certain) to be alerted to infanticidal attacks.

#### Attempted intra-community infanticides

We also considered the failed attempts to commit infanticide and attacks on the mother with the potential to cause infant death, but in which the infant survived (i.e., severe, prolonged physical attacks on mothers who were currently holding infants). There were nine such cases. In four of these nine cases, an attempt to snatch the infant was explicitly recorded. One of these four infants was killed at a later date. Of the five infants whose mothers were attacked but no clear attempt on their own life was made, three were later killed. We therefore considered all these attacks to be attempted infanticides, regardless of whether an attempt to take the infant was observed.

#### Defense of infants

On several occasions, records indicated that third parties provided some form of defense for the mother and infant. We excluded cases in which observers only attributed motivations to individual chimpanzees but did not provide behavioral details (for example: simply stating that X wanted or tried to defend). We retained cases in which named individual chimpanzees were recorded as either attacking the infanticidal individual(s) or chasing them away. In one instance an individual who initially acted as a ‘protector’ subsequently attacked the mother himself; we did not include this as a case of defence, because even if the male was initially motivated to defend the mother and infant, the outcome of the interaction was an attack.

## Results

Example one: Male-committed infanticide

*Date: 08/04/2016*

*Adult female Irene is in a party with three other adult females. She has a 6*-*day-old male infant. At 10:30, adult male Frank joins the party and approaches Irene. She pant-grunts to him. A few minutes later, Frank moves closer and Irene pant-grunts again. At 10:45, Frank chases Irene into the undergrowth and emerges holding her infant, followed immediately by Irene, who is screaming at him. The infant’s belly is ripped open. At 10:50, Frank begins to eat the infant. After a few minutes, he swings the carcass against the undergrowth and throws it from him. No other individuals intervened or became involved.*

*Observers: Geresomu Muhumuza, Charlotte Grund*

Example two: Female-committed infanticide

*Date: 06/09/2013*

*Observers hear screaming at 15:25. On investigation, they find a party containing two adult females, an adolescent female and two adult males. There is lots of blood and pieces of flesh on the ground. Night, the adolescent female is dragging a newborn male infant along the ground. The infant is still alive at this point. The infant dies soon after, by around 15:30. Night and her mother sit with the carcass and act protectively, barking at other individuals when they approach. An hour later, at 16:30, Night and her mother leave. For 10* *min from 16:30, another adult female, Mukwano, feeds on the carcass.*

*Observers: Monday Gideon, Geresomu Muhumuza, Chandia Bosco, Caroline Asiimwe*

Example three: Male-committed infanticide; attempted defense by female relative

*Date: 27/09/2017*

*At 14:30, alerted by vocalizations, observers come across a party of five adult females, an adolescent female, and one adult male. The adult male, Musa, is attacking Ramula, a young natal female who is holding a new*-*born infant of undetermined sex. Ramula is attempting to shield the infant and Musa is repeatedly beating her on the back and attempting to grab the infant. The baby has a large head wound; much of the scalp has been ripped off. Ramula’s mother, Ruhara, gets between Musa and Ramula and attacks Musa. Musa’s mother Nambi becomes involved, screaming and attacking Ramula. The adolescent female approaches Ramula and is bitten by Nambi. The attack dies down and Ruhara sits between Musa and Ramula. The infant son of one of the other females picks up pieces of the victim’s scalp from the ground and plays with it. Other individuals approach the group and as Musa looks towards them, Ramula runs away. She is followed by several adult males who have just arrived. They all surround her and look at the baby, which is still alive and whimpering. Ramula then drops the baby, which is retrieved by her mother, Ruhara. They travel together and observers lose sight of them.*

*Observers: Adriana Lowe, Geraldine Ischer, Monday Gideon*

### Frequency of attacks

**Fig. 1:**
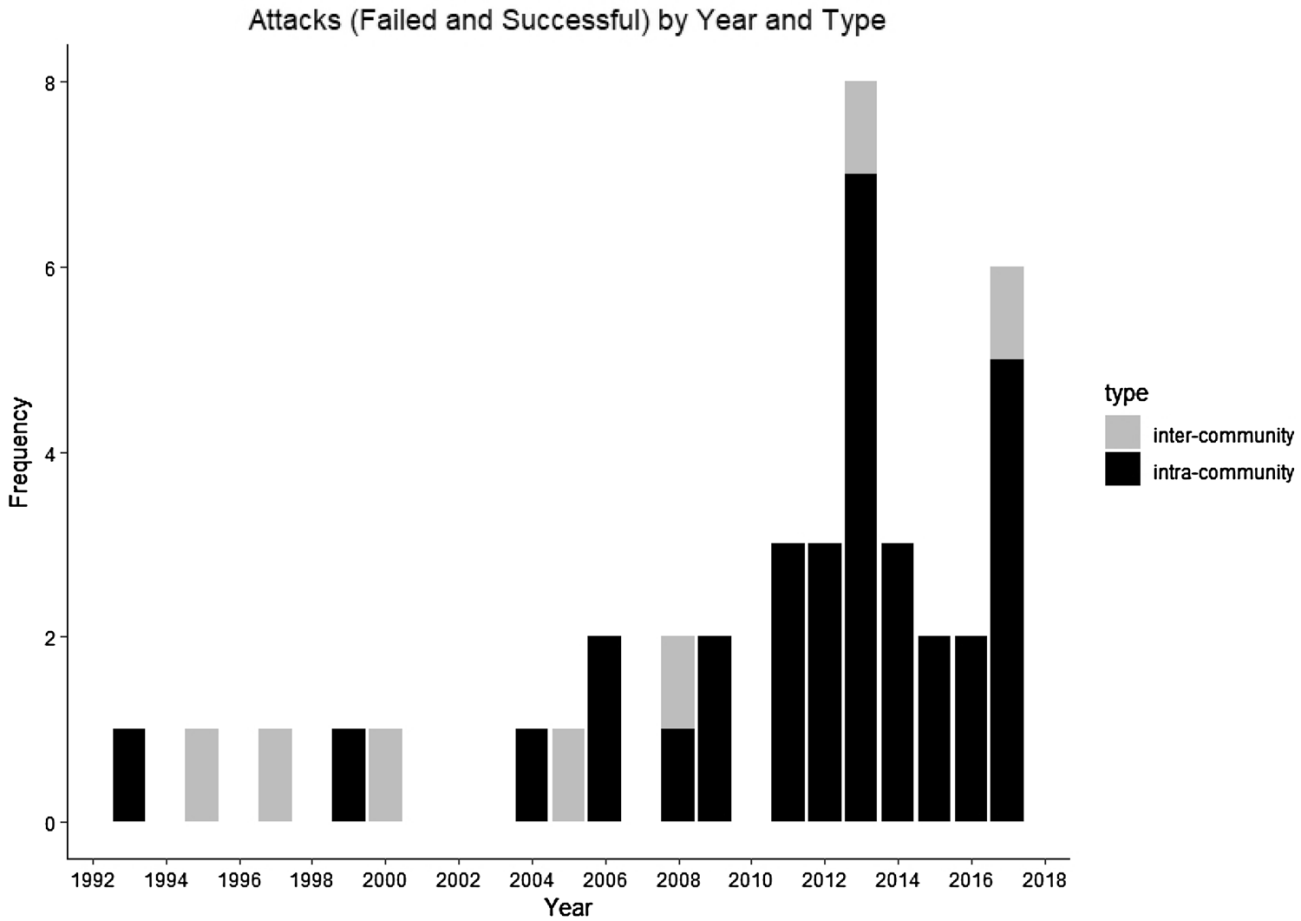
Attacks (failed and successful) by year and type (intra-community and probable or confirmed inter-community). Inter-community attacks are not included in subsequent analyses and are shown here only to illustrate the total number of infanticides recorded for this community

Of the 46 infant deaths (Table 2), three were not due to infanticide, while for 11 we had no record of any cause of death. Of the remaining 32 deaths, five were probable inter-community infanticides, three were ambiguous (for example: Sonso community individuals found with an infant chimpanzee carcass in the periphery of their home range), and 24 were candidates for intra-community infanticides. It was not always clear from the records whether an attack was inter- or intra-community when it was not observed directly, so we used location to infer in some of these cases, i.e., attacks that occurred inside the community home range were assumed to be intra-community attacks, even if it was not obvious to which female the infant belonged. Of these 24 deaths, 11 were definite intra-community infanticides, four were ‘almost certain’, and nine were ‘suspected’ (Table 2). On average, the Sonso community saw one intra-community infanticide per year (range, 0–7). Hereafter, we consider these together with the nine failed attempts (above), and refer to them throughout as ‘attacks’: 33 cases (on 30 victims, since some individuals were victims of failed attacks then subsequently attacked again on a separate occasion). Peaks in infanticidal behavior occurred in 2013 and 2017, which saw seven and five attacks, respectively (Fig. 1).

**Table 2:** Categories of infant deaths/disappearances for the Sonso community, together with the sex of attacker (where relevant), 1993–2017

	Definition	#	Sex of attacker			
			Male	Female	Both	Unknown
Definite	Kill, fatal wounding, or separation from mother observed	11	9	1	1	0
Almost certain	Attack observed, infant subsequently died or disappeared	4	2	2	0	0
Suspected	Evidence points towards infanticide (injuries, behavior of mother/other individuals)	9	5	1	0	3
Non-infanticidal	Evidence for alternative explanation	3	n/a	n/a	n/a	n/a
Unknown	No evidence	11	n/a	n/a	n/a	n/a

### Age and sex of victims of attacks

**Fig. 2:**
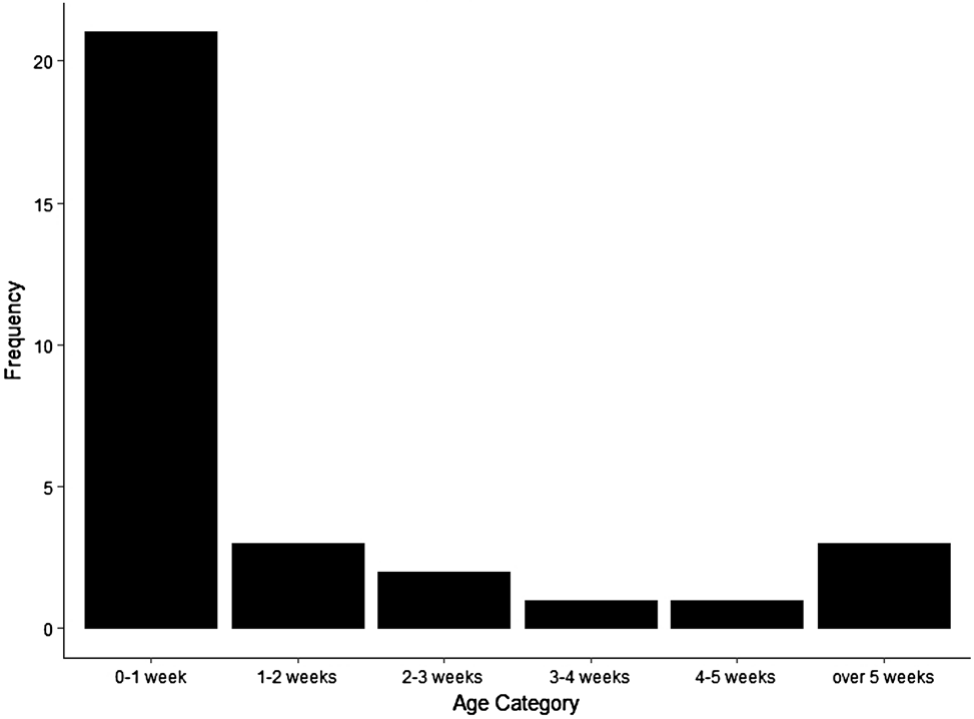
Number of attacks (failed and successful) by age category of the victim

In general, victims of attacks were very young (Fig. 2). All but three incidences occurred when infants were under 5 weeks old and two-thirds of the attacks for which the age of the victim was known were on infants under 1 week in age. Excluding two infants whose age was unknown, victims had a mean age of 29 (± 85) days. There were three outliers where the targeted infants were aged 120, 180, and 439 days. Excluding these, the mean age of victims was six (± 8) days. Of the 33 attacks, 11 of the victims were female, eight were male, and 11 were unsexed (30 victims total despite 33 attacks as three infants survived only to be killed in a subsequent attack). The difference in the number of male and female victims was not significant (exact binomial test: *p* = 0.58). Infanticidal males did not preferentially target male infants (adult male only attacks where infant sex was confirmed: ten female infants, five male infants: exact binomial test *p* = 0.67) and female-only attacks with victims of confirmed sex were too rare to identify any trend (one female victim, two male victims).

### Cannibalism

In 19 cases, observers knew whether infants were cannibalized either because they saw the attack or subsequently saw the carcass. Cannibalism was observed in five cases (26%): in three of these, the attacker(s) cannibalized the victim, in one case an individual who may or may not have been the attacker cannibalized the victim, while in the remaining case individuals who were not involved in the attack cannibalized the victim. Cannibalism was considered probable in one further case (due to the absence of some body parts), giving an estimated cannibalism incidence of 32%. In most cases, records did not describe the amount of the carcass that was eaten, but specific body parts, e.g., a limb, were mentioned, indicating that the whole corpse was not always consumed. In one case, the fingers were eaten and the belly bitten into, while in another, cannibalistic individuals moved off “leaving only the head behind”, but it is not clear whether all other parts were cannibalized, were taken by the individuals, or simply scattered and left behind. Mothers and maternal kin of the victims were not observed cannibalizing their kin.

### The mother

Mothers whose infants were subject to attacks were aged between 14 and 39 years (median 21 years, mode 15 years). Primiparous mothers lost 12 infants, while infants later in the birth order were attacked less frequently (Table 3). Of the 37 Sonso community females recorded giving birth since 1993, six were natal females. Of the 33 attacks, ten were on natal females and their offspring, 22 were on non-natal females, and in one case the mother was unidentified. Despite only 8% of births being from natal mothers, and their infants making up 30% of victims, there was no difference in the likelihood of losing an infant to infanticide for natal and immigrant females. The difference in the proportion of natal females in the community overall and the subset of females whose infants were attacked was not significant (two-sample proportion test: *p* = 0.09).

**Table 3:** Targeting of infanticidal attacks (successful and unsuccessful) by birth order of victim

Birth order of victim	Frequency
Unknown	2
1	12
2	4
3	3
4	4
5	6
6	1
7	1

Excluding females who lost infants during the final year of the study period (as these individuals would not have had time to reproduce again within this period), 89% went on to successfully give birth again within the community. The average time from infant death to next conception (calculated by subtracting 225 days, the average gestation period for this subspecies (Wallis 1997) from the birth date of the subsequent infant), was 241 days; considerably lower than the average interbirth interval in this community when infants survive (1907 days: Emery Thompson et al. 2007b).

Some infanticidal attacks involved considerable aggression towards the mother, with the mother suffering visible injuries in ten of the 33 attacks. Injuries included bleeding and limping but nothing visibly more serious. In the remaining 23 cases, mothers sustained no visible injury. No mother died as a result of one of the attacks and no females disappeared in the immediate aftermath of an attack.

### The attackers

Unfortunately, the carcass of infants who died through infanticide could only rarely be recovered limiting the availability of samples for paternity analysis. Paternity of the victim and paternity of the mother’s next infant is not known for any of the cases in which the identity of the attacker was recorded.

Not all community members were equally likely to commit infanticide. Any individual who attacked the mother/infant, or who carried or fought over the infant while it was still alive, was categorized as ‘involved’. Inspection of the infant (peering at, sniffing), before or after death, was not counted as involvement, nor was interacting with the carcass (touching, carrying). Not all individuals ‘involved’ were killers or were necessarily involved in the initial attack, but by carrying or fighting over victims while they were still alive they will have contributed to either their injuries, or their continuing separation from their mothers, and are therefore considered to have played a non-insignificant part in victims’ deaths.

Infanticide was a more common behavior for males than for females: 12 (of 34) adult males were involved in at least one attack, whereas only five (of 50) adult females were involved (Table 4). The sex of attackers was known in 20 cases (11 definite infanticides and nine failed attacks). Where the attacker(s)’ identity was not certain, we categorized the case either as ‘probable’, if it was not conclusive, e.g., the early stages of the attack were observed but the actual killing was not observed or an individual was seen holding the victim after the attack, ‘suspected’, if certain individuals had previously attacked or harassed the mother, or ‘unknown’. Males were observed, probably, or suspected to be involved in 23 occurrences, females in four, and both sexes in three. For three cases, the sex of the attacker was unknown.

**Table 4:** Identity codes and sex of individuals involved in infanticidal attacks (some individuals involved in multiple attacks)

ID	Sex	Number of attacks
FK	Male	5
HW	Male	6
NK	Male	5
MS	Male	4
SM	Male	3
MG	Male	1
JM	Male	1
KZ	Male	1
PS	Male	1
ZL	Male	3
SQ	Male	1
KT	Male	1
HT	Female	2
NB	Female	4
NR	Female	1
NT	Female	1
ZM	Female	1

### Defense by other community members

Some level of defense or protection of the mother/infant by other community members was record in five cases (male protector = two cases; female protector = three cases). In 13 cases, the attack was not clearly seen, and in 15 cases there was no observed defense by individuals other than the mother. Of the two cases where an adult male provided defense, one involved the adult male FK defending his mother FL and her infant from attack by multiple other adult males, while in the other, the adult male KT chased SM, another adult male, who had been attacking the adult female OK and her infant. All female protectors were close maternal kin of the infant being attacked (grandmother or sibling). Two of these instances of protection were by the same female, RH. Two of her daughters (RS and RM) remained in the Sonso community to reproduce, and experienced four attacks on their offspring: RM’s infant was killed, two of RS’s infants were victims of failed attempts and one of these was later killed. RH was present for two of the four attacks and in both cases, despite being an estimated 48 and 52 years old, respectively, she physically defended the infants from attack, blocking the attacker’s path and chasing attackers away. The third female to receive kin support was KY, a mother whose daughters (15 and 6 years old) attacked and injured one of the adult male attackers. The females who received support from female kin were all natal females, and kin intervened in three of the five attacks on natal females that were clearly observed. There were also occasions in which behavior which subjectively looked like defense to observers (vocalizations, standing between attackers and victims) was observed. These were considered too ambiguous to be included, although we accept that because of this, we may have underestimated the frequency of defense by other community members.

## Discussion

Infanticide was the most common cause of infant death in the Sonso community, accounting for 63% of total infant mortality. Of 103 known births, 23% became victims of infanticide. Even a more conservative estimate that excludes suspected cases still accounts for 39% of infant mortality, with 15% of all infants killed by intra-community conspecifics. Infanticide is therefore likely to be a major selective pressure on this population: if an infant is killed at 1 week post-birth, infanticide results in a loss of 473 days of the mother’s reproductive career (summing gestation, lactation, and post-infanticide delay to conception), and the associated energetic investment, with these costs steadily increasing the longer the infant survives prior to the infanticide. These fitness costs are quantifiable. Female chimpanzees give birth on average every 5–7 years (Emery Thompson et al. 2007b), and average expected reproductive lifespan for Sonso females is roughly 26 years, given an average age at first conception in East African chimpanzees of 14.2 years (Walker et al. 2018b) and an oldest age for a live birth by a Sonso female of 40.6 years (Emery Thompson et al. 2007a, b). The most fecund Sonso female had eight recorded births, with four surviving infants, losing infants at 1 week, 1 week, 1 day, and 65 days post-partum, a total loss of 1944 days to gestation, lactation, and delay to conception. These losses constitute 20.7% (5.3 years) of this female’s 26-year reproductive career, sufficient time for an additional infant. While not all of these fatalities are confirmed to be the result of infanticide, applying the average figure of 65% attributes a loss of approximately 13.5% (3.4 years) of the reproductive career, and 2.6 live-born infants, to infanticide. Despite such costs, active defense against infanticidal attacks was rare, even though it appears to be an effective strategy: in our dataset, several attempted infanticides were prevented by the intervention of other chimpanzees, and of the nine thwarted attempts, six of the targeted infants survived infancy. Low levels of kinship are likely to be responsible for the scarcity of defense: one of the two instances of active defense by adult males, and all instances of defense by females were of natal females and their infants by close maternal kin. The typical pattern of female dispersal means that female bystanders are unlikely to be closely related to the targeted infant, while low paternity certainty in this community (Newton-Fisher et al. 2010)—even for an alpha male—is similarly likely to deter males from actively defending infants.

The evidence from the Sonso community suggests that the majority of infanticidal attacks can be explained by the sexual selection hypothesis (Hrdy 1979; Sommer 1987; van Schaik 2000). Most attacks were by males, there was no significant sex bias in victims, and mothers were not seriously injured. Two-thirds of the targeted infants were under 1 week of age, and females who lost an infant to infanticide conceived on average around seven times more quickly than if their infant had survived, with the majority of mothers who lost infants going on to reproduce again within the community. Infanticide therefore created the predicted reproductive opportunities for male attackers and the majority of the predictions of this hypothesis were met (see Table 5 for a summary of which predictions were met for each of the hypotheses). We were unable to test whether infanticidal males refrained from targeting their own infants, or whether they fathered replacement infants due to a lack of paternity data for both victims and subsequent infants born to their mothers. Victims’ carcasses were only rarely recovered and sampling for DNA was not available in early cases.

**Table 5:** Summary of the predictions of the four hypotheses for infanticide

	Sexual selection	Resource competition	Meat acquisition	Mate competition
Age bias in victims	✓	–	✗	–
Killers should not target their own offspring	Unknown	–	–	–
Killers should sire replacement offspring	Unknown	–	–	–
Sex bias in attackers	✓	✗	✗	✓
Sex bias in victims	✓	✓	✓	✗
Mothers should be uninjured	✓	–	–	–
Victims will be cannibalized in full	–	–	✗	–
Killers should eat the carcass	–	–	✗	–

*Ticks* and* crosses* indicate the predictions that were supported or otherwise by the evidence

Attacks on first-born infants were more common than those on later-born offspring, and targeting infants of primiparous females may also be driven by sexual selection. If low-ranking males are more likely to father such infants because high-ranking males have higher mating success (Tutin 1979; Nishida 1983) and males prefer mating with older, parous females (Muller et al. 2006), such infants will face increased risks because higher-ranking males will have been less likely to sire them and have greater chances of siring any replacement infant. Further, sexually selected infanticide can be considered as a form of male sexual coercion (Smuts and Smuts 1993; Muller et al. 2009) and coercive infanticide of young females has been implicated as fundamental in shaping reproductive decisions by female gorillas (Watts 1989b; Harcourt and Greenberg 2001; Yamagiwa et al. 2009). The targeting of primaparous females by male chimpanzees may therefore be an extreme example of intimidation (sensu Clutton-Brock and Parker 1995) of females at the beginning of their reproductive career in order to generate subsequent mating biases that favor infanticidal males.

The meat acquisition hypothesis (Hrdy 1979) is an unlikely explanation for infanticide in this community. Cannibalism occurred in a minority of cases and any consumption of carcasses was incomplete. Nevertheless, infanticide victims offer a source of valuable protein and micronutrients, so cannibalism may be nutritionally beneficial. Whether meat acquisition is a secondary selective force shaping infanticide or a by-product remains to be determined. If a secondary selective force, levels of cannibalism should vary inversely (at a community level) with hunting, and at an individual level with access to meat, whereas the reverse is predicted by the by-product hypothesis. A comparison between Sonso and Kasakela (Gombe) supports the idea of a by-product explanation: Sonso chimpanzees are relatively poor hunters, even though historically low levels (Newton-Fisher et al. 2002) have risen in recent years (Hobaiter et al. 2017), whereas Kasakela chimpanzees hunt more frequently (Stanford et al. 1994; Gilby et al. 2006). Cannibalism is low for Sonso, but in Kasakela, all of the victims of infanticide are cannibalized, at least in part (infanticide victims are eaten less completely than prey items: Kirchhoff et al. 2018). Thus, chimpanzees accustomed to eating meat appear more likely to consider the victims of infanticide as a suitable food—they may have more of a generalized ‘taste for meat’.

The male mate competition hypothesis was also not supported by our data, and we would argue that the slow life histories of chimpanzees makes this an unlikely hypothesis. Males reach adulthood at around 16 years (Goodall 1986), and while male reproductive careers can start at early as 10 years old in this community and continue to 47 years old, the mean ages of first and last reproduction are 16 and 25 years, respectively (Hobaiter, unpublished data). Males who survive until adulthood in the Sonso community live to an average of 29 years (comparable to those in other communities: Hill et al. 2001), making reproductive overlap between generations rare. An infant is unlikely to be a significant reproductive competitor for any males who are adults at the time of his birth. In addition, males are allies when it comes to inter-community aggression, and communities with fewer males are at a disadvantage in maintaining territories and the associated resources (Wilson et al. 2001, 2014). Killing potential future allies (even if for their own sons) would therefore seem maladaptive.

The resource competition hypothesis was not supported as a general explanation for infanticide in this community. However, a version of this hypothesis may be a plausible explanation for female-committed infanticide (while the other hypotheses are not). The potential for competition between females is high in chimpanzees (Pusey and Schroepfer-Walker 2013), and the function of infanticide may not be to remove a competitor directly but to drive away the mother (and by extension her future offspring), protecting the attacker’s resource base and thus her future reproductive success (as indeed seems the case for the Belding’s ground squirrels: Sherman 1976). Such female-committed infanticidal behavior could therefore be the product of sexual selection, as with male infanticide, as it would be the consequence of intra-sexual competition (Clutton-Brock 2007). Under this hypothesis, rates of female-committed infanticide should vary with levels of female competition, and infanticide should result in greater reproductive success for the attacker(s). Rates should increase with local population density, declining food supply, and if females are unable to exclude others from their core feeding areas. Conversely, rates should be low where food is abundant and thus female competition reduced. Sonso females experience high local population density, but this is facilitated by typically high levels of food availability (Newton-Fisher et al. 2000), including consumption of figs associated with increased reproductive rates in chimpanzees (Emery Thompson et al. 2017), and have inter-birth intervals significantly shorter than the average for other communities (based on data from Emery Thompson et al. 2007a, b). Thus, competition between Sonso females may be generally low, which could explain the very few occurrences of female-committed infanticide in our dataset. It is notable that the female-committed infanticides in this community reported by Townsend et al. (2007) occurred during a period of substantial female immigration and thus increased female competition. Future work should test the relationship between resource availability, female competition, and occurrence of infanticide to develop a parallel model of sexually selected infanticide for females. We have too few observations to draw any specific conclusions, but pooling data across study sites might be instructive.

While we cannot discount the possibility that our dataset underestimates the frequency of female-committed infanticide, female chimpanzees of the Sonso community have been followed regularly since the mid 1990s (Fawcett 2000) and are relatively gregarious (median female party size = 3.0, range, 1–14: Newton-Fisher, unpublished data). Observed opportunities for infanticide are not taken, and adult natal females with potential coalitionary support from their mothers were never seen committing infanticide, despite such support reducing the costs associated with overpowering a mother and killing the infant (cf. Goodall 1977). If female-committed infanticide is biased towards peripheral locations because more socially dominant females force younger and more subordinate females towards the periphery (as suggested for other communities: (Williams et al. 2002; Emery Thompson et al. 2007c), such attacks may be missed. However, the pursuit of peripheral females does not match the patterns of restricted ranging shown by non-cycling females (Bates and Byrne 2009), and seems counter-intuitive: females should be targeting others who are attempting to settle in their more central core areas: peripheral females have already been dissuaded from doing so. Observer expectation may also bias against recording female participation: in one case in our dataset, field assistants suspected that an adult female was involved, but the report provides no further detail; in another, several females were found sniffing the corpse of an infant, but no information was provided regarding whether these females were involved in its death. Cases with unknown attackers may also have had female perpetrators. However, with these caveats in mind, we remain confident that the qualitative difference we report in the likelihood of an attacker being male, rather than female, is representative of the reality for this community, even if our dataset may have missed some instances of infanticide.

It is not yet clear why the Sonso community appears to experience higher rates of infanticide than other communities for which data are available. While tempting to ascribe the increase in the number of recorded attacks in recent years to increased research intensity and habituation of the chimpanzees, habituation levels from the mid-1990s onward were sufficient for detailed studies of both male and female behavior, and both are high for other communities that report substantially lower rates. Inter-community infanticides were observed at Sonso in the early years of the study period when intra-community attacks were rare, further suggesting that the lower number of attacks prior to 2011 were not due to a lack of observation opportunities. The study site with the next highest number of intra-community infanticides, Gombe National Park, reported 16 intra-community infanticides during a 49-year study period (1965–2014), or 0.33 per year, across two study communities (Wilson et al. 2014), while we report here 24 over a 24-year study period with one study community, an average rate of 1.00 per year. Assuming infanticide in chimpanzees is largely a consequence of sexual selection on males, variation in rates of infanticide between communities should be due to differences in reduction of the interbirth interval (IBI), in the *B* in van Schaik’s (2000) model, or in the chance of siring a replacement infant minus the chance of having sired the existing one (*P*_*i*_*−p*). The high rates of infanticide in Sonso are unlikely to be due to a more pronounced reduction in IBI compared to other communities: in a review of data from six communities, Emery Thompson et al. (2007a) found that the reduction in IBI following infanticide was smallest for Sonso females. Instead, the difference may be due to variation in the change in likelihood of siring the replacement infant vs. the current infant (*P*_*i*_ − *p*). Since male rank predicts paternity in East African chimpanzees (Constable et al. 2001; Inoue et al. 2008; Wroblewski et al. 2009; Newton-Fisher et al. 2010; Langergraber et al. 2013), changes in rank will create variation in *P*_*i*_ − *p.* A male rising in rank increases *P*_*i*_ and, where hierarchies are steep (Kaburu and Newton-Fisher 2015b) and high-ranking males effectively limit lower-ranking males’ mating access, a male who rises rapidly in rank will experience a large increase in *P*_*i*_ relative to a small value for *p*, and thus have a high resultant *P*_*i*_ − *p*. Some level of hierarchy instability is therefore necessary to create the conditions for sexually selected infanticide to be adaptive (if males’ ranks are static, their individual average probabilities of siring infants remain unchanged), so rates should be highest where the male hierarchy is both unstable and steep. The high rates of infanticide at Sonso suggest that these males experience steeper or more unstable hierarchies than do those in other communities, and while this has been the case for certain periods, hierarchy steepness changes within communities over time (probably due to shifting male demographics: Kaburu and Newton-Fisher 2015a). Unfortunately, we do not have the necessary long-term data on male hierarchy to test whether variation in infanticide rates correlates with hierarchy steepness, and there are two caveats to any conclusion that variation in hierarchy steepness and stability explain the high rates at Sonso. First, if males do not encounter an infant at its most vulnerable, any opportunity to kill it will be substantially reduced, regardless of variation in the probabilities of paternity. Female chimpanzees use male avoidance as a counter strategy to infanticide (Lowe et al. 2018), albeit one constrained by their need to visit food patches from which they are unable to evict males. For the Sonso community, the small home range and consequent high local population density may result in infants being exposed to potentially infanticidal males more frequently than those in other study communities, particularly if females in other communities are more strongly dispersed. Second, elevated rates at Sonso as compared to other communities may be a partial artifact of general under-reporting: as relatively infrequent behaviors, the corpus of observations from a single community may not be regarded as significant enough for publication (cf. Wilson et al. 2014). Alternatively, observing infanticides may be more difficult where population densities are lower, home ranges are larger, and/or females are less gregarious.

In summary, our analysis of the largest dataset on infanticides from a single community shows that this behavior is a major cause of infant death and a significant selective pressure. The majority of infanticides are committed by adult males, targeting very young infants, and are best explained by the sexual selection hypothesis. Sonso chimpanzees are less cannibalistic than those of other study communities, and this behavior appears to be a by-product of infanticide, at least for this community. Infanticide by females was rare, but we suggest that this may also be the consequence of sexual selection, in this case, intra-sexual competition between mothers. We suggest that the high rates of infanticide in the Sonso community may be due to a possible combination of steep, unstable male hierarchies, and the difficulties mothers of young infants have in avoiding potentially infanticidal males due to the small home range. However, infanticide is under-reported and attempted infanticides even more so, and infanticide may be more prevalent across chimpanzee communities than previously thought.

Note: Since the completion of this study, two further intra-community infanticides and one further attempted infanticide have been reported from the Sonso community (E. Holden & A. Soldati, personal communication).
